# Postnatal maturation of endogenous opioid systems within the periaqueductal grey and spinal dorsal horn of the rat

**DOI:** 10.1016/j.pain.2013.09.022

**Published:** 2014-01

**Authors:** Charlie H.T. Kwok, Ian M. Devonshire, Andrew J. Bennett, Gareth J. Hathway

**Affiliations:** a Laboratory of Developmental Nociception, Queens Medical Centre, University of Nottingham, Nottingham NG7 2UH, UK; b FRAME Laboratory, Queens Medical Centre, University of Nottingham, Nottingham NG7 2UH, UK

**Keywords:** μ-Opioid receptors, Development, Neonate, Periaqueductal grey, Dorsal horn

## Abstract

Significant opioid-dependent changes occur during the fourth postnatal week in supraspinal sites (rostroventral medulla [RVM], periaqueductal grey [PAG]) that are involved in the descending control of spinal excitability via the dorsal horn (DH). Here we report developmentally regulated changes in the opioidergic signalling within the PAG and DH, which further increase our understanding of pain processing during early life. Microinjection of the μ-opioid receptor (MOR) agonist DAMGO (30 ng) into the PAG of Sprague-Dawley rats increased spinal excitability and lowered mechanical threshold to noxious stimuli in postnatal day (P)21 rats, but had inhibitory effects in adults and lacked efficacy in P10 pups. A tonic opioidergic tone within the PAG was revealed in adult rats by intra-PAG microinjection of CTOP (120 ng, MOR antagonist), which lowered mechanical thresholds and increased spinal reflex excitability. Spinal adminstration of DAMGO inhibited spinal excitability in all ages, yet the magnitude of this was greater in younger animals than in adults. The expression of MOR and related peptides were also investigated using TaqMan real-time polymerase chain reaction and immunohistochemistry. We found that pro-opiomelanocortin peaked at P21 in the ventral PAG, and MOR increased significantly in the DH as the animals aged. Enkephalin mRNA transcripts preceded the increase in enkephalin immunoreactive fibres in the superficial dorsal horn from P21 onwards. These results illustrate that profound differences in the endogenous opioidergic signalling system occur throughout postnatal development.

## Summary

The increase in expression of endogenous opioidergic targets coincides with functional maturation of pain signalling in the third postnatal week of the rat.

## 1 Introduction

Supraspinal modulation of spinal pain processing involves sensitive and precise orchestration of signalling between distinct anatomical regions, particularly in the periaqueductal grey (PAG) and nuclei of the rostral ventromedial medulla (RVM) [50]. These areas form part of a descending pain control axis, with the PAG integrating pain-related information from the forebrain and relaying appropriate neuromodulatory information to the dorsal horn (DH) of the spinal cord via pro- and antinociceptive descending pathways arising in the RVM [5], [19], [24].

Neonatal responses to noxious stimuli are exaggerated and often inappropriate [3], [17], [22], [48]. The differences between mature and neonatal noxious processing are thought to be underpinned by increased excitation and decreased inhibition at the level of the DH [28]. Supraspinal control of spinal nociceptive reflexes is slow to develop over the postnatal period [29]. We have previously shown that although both descending facilitation and inhibition of spinal excitability can be evoked in adults when the RVM is electrically stimulated, in neonatal and juvenile rats, only descending facilitation can be evoked until at least postnatal day (P)28 [28].

It is known that pharmacological activation of μ-opioid receptors (MORs) within the adult descending pain modulatory pathway, particularly the PAG and RVM, results in potent analgesia [5], [6], [18], [19], [20], [46], [53], [57]. Previously, we have shown that microinjection of the MOR agonist [D-Ala_2_, N-MePhe_4_, Gly-ol]enkephalin (DAMGO) into the RVM of lightly anaesthetised adult rats produces a dose-dependent decrease in spinal excitability, whereas the same dose of DAMGO in P21 rats produces reflex facilitation [29]. Additionally, we have demonstrated that blocking the central actions of endogenous opioid peptides with the potent opioid receptor antagonist naloxone between P21 and P28 prevents the normal development of descending RVM inhibitory control of spinal nociceptive reflexes [29].

These data indicate that the developmental transition from supraspinally mediated descending facilitation to inhibition of spinal excitability emanating from the RVM is controlled by opioidergic activity within the pain modulatory circuit during a critical period around P21. Furthermore, it suggests that there is a postnatal refinement in opioidergic neurotransmission within the central nervous system. Although existing evidence suggests that the switch in supraspinal control of spinal excitability during postnatal development may be driven by opioidergic activity within the pain modulatory circuit, much of this work has been done at the level of the RVM. A study of opioidergic activity in the PAG over postnatal development has been neglected.

In this study we demonstrate that significant refinement occurs within specific components of opioidergic systems of the PAG and DH, which has profound effects on the influences of these centres on pain processing.

## 2 Methods

### 2.1 Animals

All animal procedures were licensed by the UK Home Office and performed in accordance with the Animals (Scientific Procedures) Act 1986. Our experiments adhered to the guidelines of the Committee for Research and Ethical Issues of the International Association for the Study of Pain. Postnatal day 3, day 14, and adult (240–260 g) Sprague-Dawley rats were purchased from Charles River Laboratories (Margate, UK). Pups were housed with their dams in individually ventilated cages in an in-house animal facility, weaned when they reached P21, and then group housed in same-sex cages. Free access to food and water was available throughout. Experiments were performed on P10, P21, and P40 (adult) rats, and different cohorts of rats were used in electrophysiological, immunohistochemical, and TaqMan real-time polymerase chain reaction (RT-PCR) studies. All procedures were performed during the animals’ light cycle.

### 2.2 Surgery

PAG microinjection animals were anaesthetised with isoflurane (Baxter; Newbury, Berkshire, UK) and mounted on a stereotaxic frame (Kopf Instruments, Tujunga, CA, USA). The skull was exposed and bregma was located. Stereotaxic coordinates for the ventral PAG (vPAG) were calculated (both adult and P21: left-right [L-R] 0.5 mm; anterior-posterior −7.8 mm; dorsal-ventral [D-V] −6.0 mm; P10: L-R 0.5 mm; anterior-posterior −7.8 mm; DV −4.5 mm) and a 26-gauge 2.5-μL syringe (Hamilton, Reno, NV, USA) was inserted through a drilled hole in the skull. Drugs were injected over a 5-minute period, after which the syringe was removed and the wound was closed. Total volume of drug administered into the PAG was 1 μL; only one drug was administered per animal.

Laminectomy animals were anaesthetised and mounted on a stereotaxic frame. Laminectomy was performed to expose the L4-5 segments of the spinal cord. The dura mater was carefully removed, leaving the pia mater intact. This method allows the drug to be applied directly onto the spinal cord.

**Fig. 1:**
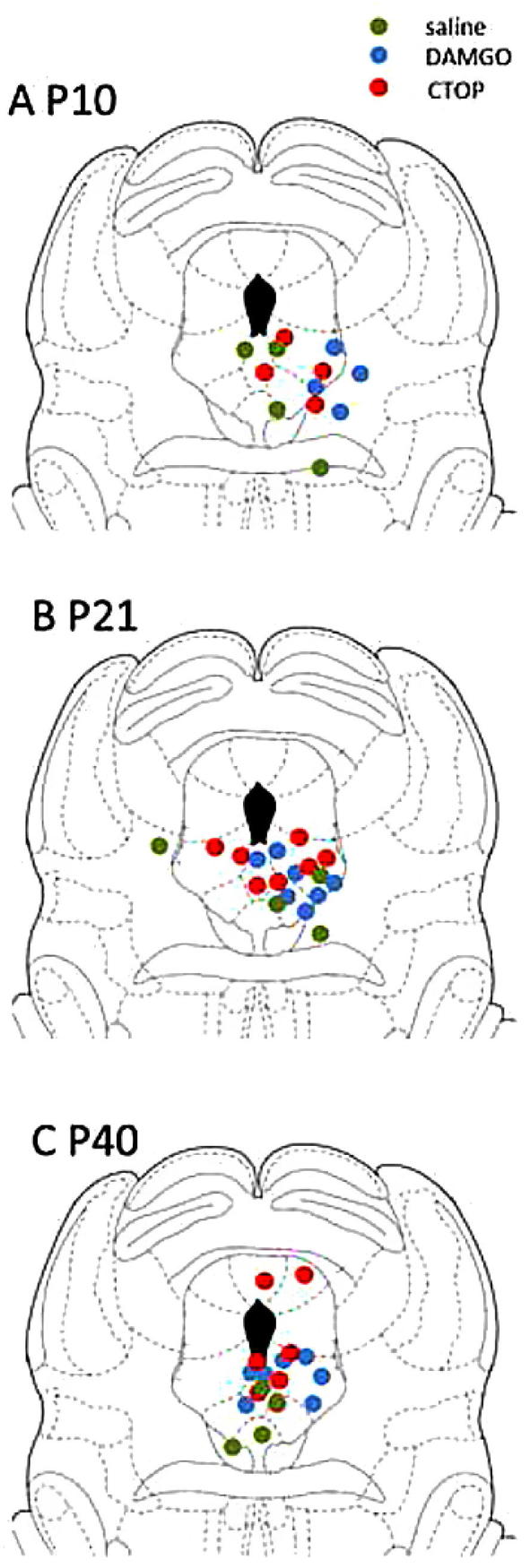
Injection sites for saline, DAMGO (30 ng), and CTOP (120 ng) were identified histologically post electromyogram recording.

Drugs DAMGO (MOR-agonist, 30 ng; Tocris, Abingdon, Oxon, UK) and CTOP (MOR-antagonist, 100 ng; Tocris) were administered at doses determined from previously published studies in adult brainstem [28]. Saline was administered in separate sets of animals as vehicle control, and it was confirmed that saline either injected into the PAG or spinally applied had no significant effect on spinal reflex excitability. The experimenter was blinded to the drug administered. Sites of injection in the PAG were confirmed by examining the lesion tracts after electromyographic (EMG) recordings (Fig. 1). The brains were quickly dissected out and kept on dry ice, and were then coronally sectioned on a freezing microtome (Leica, Milton Keynes, Bucks, UK). The lesion sites were recorded. All DAMGO injection sites lay within the vPAG. Data from 2 adult rats following CTOP microinjection were excluded because the injection sites fell outside of the vPAG.

### 2.3 Electrophysiological recording

EMG recordings were performed as described previously [24]. Anaesthesia was initially induced at 5% isoflurane, which was then reduced to 3% for surgery. After surgery, isoflurane concentration was further reduced to 1.3% to keep the animals lightly anesthetised. Anaesthesia in P21 and adult rats were maintained with a surgically implanted endotracheal cannula, whereas in P10 rats it was maintained with a fitted nose cone. Animals were mounted onto the stereotaxic frame after tracheotomy. The fur overlying the biceps femoris muscle was trimmed and a bipolar concentric needle EMG recording electrode (comprising a modified 27-gauge hypodermic needle; Ainsworth, Coventry, UK) was inserted into the belly of the muscle. Such recording electrodes ensure that recorded activity is restricted to local muscle activity in small animals. The EMG electrode was connected to a NeuroLog head-stage (NL100AK; Digitimer, Welwyn Garden City, UK), signals amplified ×2000 (NL104A), band-pass filtered between 10 and 1000 Hz (NL125) before being sampled at 2 kHz using LabChart software via a PowerLab data acquisition unit (AD Instruments Ltd., Oxford, UK). Isoflurane anaesthesia was maintained at 1.3% (small animal ventilator; Harvard Apparatus, Kent, UK) and rats were left to equilibrate to anaesthesia for 30 minutes before recording. In these experiments, spinal reflex excitability was determined by the EMG activity of flexor hind limb muscle evoked by mechanical stimulation of the plantar hind paw using von Frey hairs (vFh). The vFh is a device that measures mechanical sensitivity/threshold [13]; it is a series of fine plastic monofilaments of varying diameters that have progressively stronger bend strengths. The force required to bend each monofilament is calibrated so that measurements can be standardised from one individual animal to another.

Responses to 2 sub-threshold vFh (T-1, T-2), and the threshold hair (T) and a supra-threshold hair (T+1) were recorded and the same 4 hairs used in all subsequent stimulation conditions for data analysis. Thresholds were determined as the vFh that produced an EMG response more than 10% greater than the resting EMG activity. Each hair was then applied 3 times, and the mean reading for each of the 3 presentations recorded. Previous studies have shown that mechanical withdrawal thresholds are significantly lower in P10 and P21 than in adult animals [21], therefore, different hairs were used in each age group.

A stimulus-response curve of EMG magnitude vs mechanical stimulus intensity was plotted and the area under the curve was calculated to provide an integrated measure of spinal reflex excitability. The value obtained was considered as the predrug response of the animal. Subsequent responses following pharmacological manipulations were normalised to this because of variations in background (nonevoked) EMG activity. vFh stimulations were carried out for the next 30 minutes at 10-minute intervals. Predrug responses were normalised as 100%, therefore, an increase (>100%) indicates a facilitation, whereas a decrease (<100%) indicates an inhibition of spinal reflex excitability.

In this study, mechanical threshold postdrug administration was presented as a percentage change of the predrug threshold ([mechanical threshold postdrug administration (grams) − pre-drug threshold (grams)]/predrug threshold * 100%). Therefore, a positive value would indicate an inhibition of nociceptive responses (analgesia), whereas a negative value would indicate facilitation.

### 2.4 Tissue collection for TaqMan RT-PCR

P10, P21, and adult rats were killed by an overdose of pentobarbital (2.5 mL per animal, intraperitoneally) and brains and spinal cords were quickly dissected out on ice. Tissue from the PAG and lumbar (L3-L5) spinal cord were isolated, with the PAG and spinal cord further subdivided into dorsal and ventral parts with a scalpel blade. Samples were put into separate cryovials for flash freezing in liquid nitrogen and stored at −80°C. Three to seven animals were included in each age group.

### 2.5 Quantitative real-time PCR

TaqMan quantitative RT-PCR was performed using the StepOnePlus real-time PCR system (Applied Biosystems, Forster City, CA, USA). Primers and probes for glyceraldehyde 3-phosphate dehydrogenase (GAPDH; National Center for Biotechnology Information [NCBI] reference sequence NM_017008.3), pro-opiomelanocortin (POMC; NCBI reference sequence NM_139326.2), enkephalin (ENK; NCBI reference NM_017139.1) and MOR 1A (MOR-1A; NCBI reference sequence NM_001038597.2) were designed on Primer Express 3 (Applied Biosystems). The POMC and GAPDH probes were labelled at the 5′ end with 6-carboxyfluorescein and at the 3′ end with tetramethylrhodamine. The MOR-1A and ENK probes were labelled at the 5′ end with 6-carboxyfluorescein and the 3′ end with dihydrocyclopyrroloindole tripeptide minor groove binder. The probes were specifically designed to span across an intron-exon boundary in order to avoid potential amplification of genomic DNA in the analysed samples. Each sample was loaded onto 96-well plates and run in triplicate. Expression of target genes (POMC, MOR-1A, ENK) were normalised to GAPDH, and transcript regulation was determined using the relative standard curve method per manufacturer’s instructions.

### 2.6 Perfusion

Rats that were included in immunohistochemical investigations were perfused transcardially with 150 mL of saline and then 200 mL of 4% paraformaldehyde (Sigma, Dorset, UK) in 0.1 M phosphate-buffered saline (PBS). Immediately after fixation, brains were removed and postfixed in 4% paraformaldehyde at 4 °C overnight. Tissue was stored in 30% sucrose solution in 0.1 M PBS containing 0.01% azide (Sigma) for at least 1 day for cryoprotection before sectioning.

### 2.7 Immunohistochemistry

The PAG and lumbar spinal cord were sectioned using a freezing microtome (Leica, model number SM2010R) set at a nominal thickness of 40 μm. The free-floating sections were washed in 0.1 M PBS 3 times. The sections were blocked in 3% goat serum, 0.3% triton solution in 0.1 M PBS for 1 hour at room temperature immediately before they were incubated with primary antibodies to reduce nonspecific background staining. All antibody solutions were made up in Tris-buffered saline. Sections were incubated overnight with constant agitation at room temperature. The primary antibodies used were rabbit anti-POMC (1:100; Phoenix Pharmaceuticals Inc., Burlingame, CA, USA), rabbit anti-MOR (Neuromics, Edina, MN, USA; 1:1000 with tyramide signal amplification protocol), and mouse anti-ENK (1:100; Fitzgerald, Acton, MA, USA) overnight at room temperature. For direct staining, sections were washed in 0.1 M PBS 5 times at 10-minute intervals and incubated with fluorescent secondary antibodies for 2 hours at room temperature. Goat antirabbit Alexa-488 (Invitrogen, Paisley, UK) was used to detect POMC immunoreactivity, and goat antimouse Alexa-568 (Invitrogen, UK) to detect ENK immunoreactivity.

Following incubation of rabbit anti-MOR antibody, signals were amplified using a tyramide amplification protocol. Sections were first incubated with appropriate secondary biotinylated antibodies (1:400, 1.5 hours) followed by avidin-biotin complex (ABC elite; 1:250 Vectastain A plus 1:250 Vectastain B; Vector Laboratories, Peterborough, UK) for 30 minutes followed by a signal amplification step with biotinylated tyramide solution (1:75, 7 minutes; Perkin Elmer, Buckinghamshire, UK). Sections were then incubated with fluorescein isothiocyanate avidin (1:600, Vector Laboratories) for a further 2 hours.

Finally, after secondary antibody or tyramide signal amplification incubation, sections were washed 5 times at 10-minute intervals with 0.1 M PBS (first 4 washes, the last wash with ddH_2_O). They were then mounted on gelatine-coated slides and coverslipped using Fluoromount (Sigma).

### 2.8 Microscopy and quantification

Immunofluorescent sections were observed with a Leica IRE2 fluorescence microscope fitted with Hamamatsu Orca-ER monochrome camera and captured using Volocity 6.1 software (Perkin Elmer, UK). Image J 1.29 (National Institutes of Health, Bethesda, MD, USA) was used to adjust brightness and contrast of the images postacquisition. Three to six brains per age were processed in this way. For each anatomical region (vPAG and DH), the area of interest was outlined and selected by using the region of interest function of the Volocity software and the motorised stage (Prior Scientific, Cambridge, UK). Forty-times magnification images were taken for each section. Same exposure time of image acquisition was used for each section, staining for the different antibodies from the different animals to ensure consistent brightness in images. The 40× images (raw tiles) were then stitched together at 40% overlapping to form a composite image of the area of interest; the raw tiles were saved into a separate folder for quantification.

Systematic random sampling and unbiased stereological methods were used for quantification as adopted from previously published studies [26], [37]. Six raw tiles were chosen from area of interest for quantification, the first raw tile was always included, and 5 other raw tiles were selected at regular intervals according to the number of tiles collected for the section. Number of cells and staining intensity were accounted for as follows. A counting frame was superimposed onto the raw tile, the right and upper boundary of the tile were “forbidden lines,” the left and bottom boundary were “acceptance line.” Number of cells was counted only if they either lay entirely within the counting frame or cross an acceptance line without also crossing a forbidden line [31], [37]. The staining intensity was estimated with Image J. The data obtained were added together to form an estimate of the number of POMC-, MOR-, and ENK-positive cells or fibres. This process was repeated for each animal.

For the purpose of presentation, images were also captured on a Leica DMIRE fluorescent microscope with a TCS SP1 confocal head and a 40× oil immersion objective.

### 2.9 Data analysis and statistics

All individual data points were represented as mean ± SEM. EMG data were normally distributed. Comparisons were made between the baseline values and those obtained following intra-PAG microinjections within each individual animal using repeated-measures one-way analysis of variance (ANOVA) with Bonferroni posttests. Statistical comparisons between the age groups and drugs were made using 2-way ANOVA with Bonferroni posttests. Statistical comparison between the age groups for the expression of various endogenous opioid targets in TaqMan RT-PCR and immunohistochemical experiments were made by one-way ANOVA with Bonferroni posttests.

## 3 Results

### 3.1 Baseline EMG activity does not change significantly between ages

**Fig. 2:**
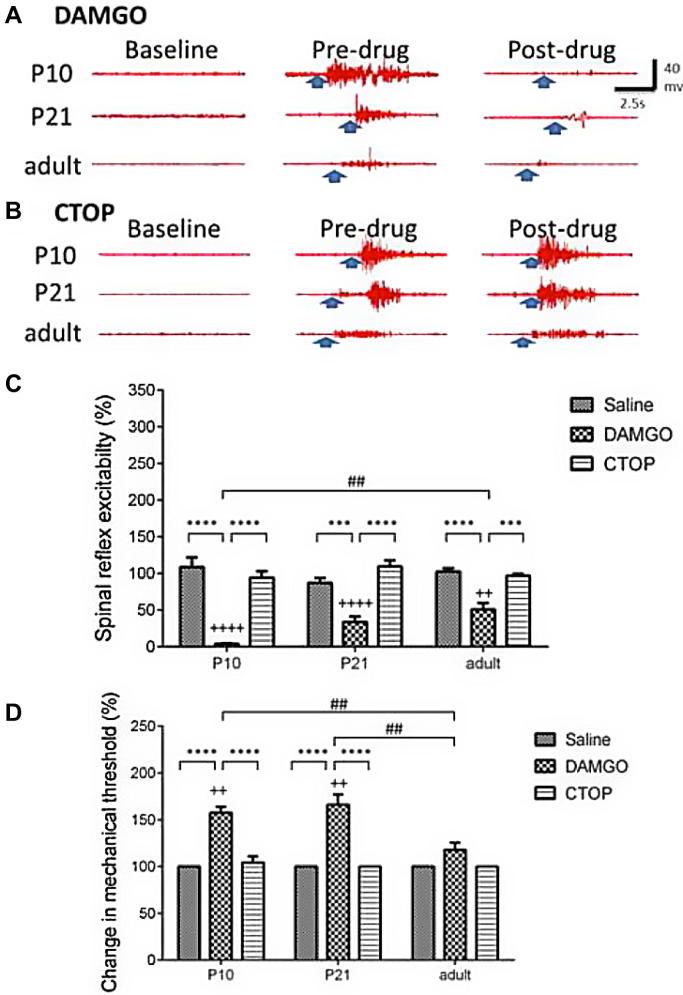
Spinal μ-opioid receptor (MOR) activation reduces nociceptive responses in all ages. (A) Typical electromyographic (EMG) traces in postnatal day (P)10, P21, and adult rats during baseline (without any stimulation), predrug (a typical threshold response before application of drug) and postdrug (A: DAMGO 30 ng; B: CTOP 120 ng) periods. The application of MOR opioid agonist DAMGO onto the spinal cord produced antinociceptive responses in all ages tested. Spinal reflex excitability (C) was decreased when compared to age-matched saline and predrug responses in all ages, both saline and CTOP had no significant effect. Mechanical threshold (D) was increased when DAMGO was administered in P10 and P21 rats. ^++^, ^++++^*P* < 0.01, 0.0001 compared to predrug responses. ^∗∗∗^, ^∗∗∗∗^*P* < 0.001, 0.0001 compared to other age-matched drug groups. ^##^*P* < 0.01 compared to other drug-matched age groups.

**Fig. 3:**
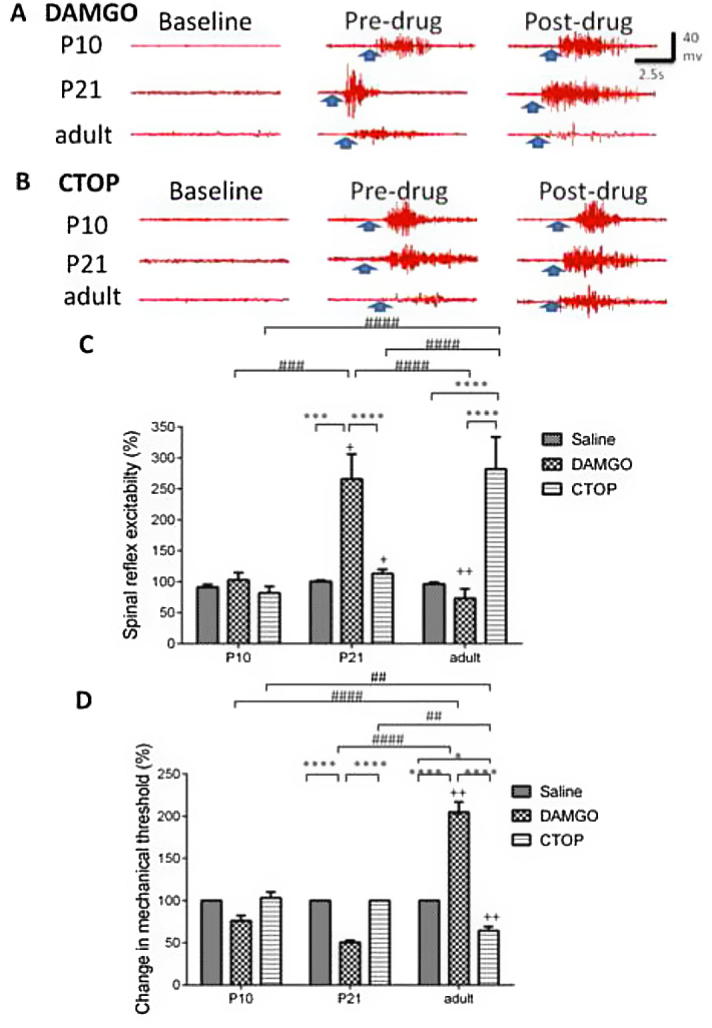
Periaqueductal grey (PAG) μ-opioid receptor (MOR) activation produces differential age-specific effects in nociceptive responses. (A) Typical electromyographic (EMG) traces in postnatal day (P)10, P21, and adult rats during baseline (without any stimulation), predrug (a typical threshold response before application of drug) and postdrug (A: DAMGO 30 ng; B: CTOP 120 ng) periods. The microinjection of DAMGO and CTOP into the PAG produced differential nociceptive responses between the ages. Spinal reflex excitability (C) post-DAMGO was unchanged in P10 rats, but was facilitated in P21 and inhibited in adults when compared to predrug responses and to age-matched saline groups. CTOP elevated spinal reflex excitability in P21 rats when compared to predrug responses, and in adults when compared to age-matched saline groups. Mechanical threshold (D) was unchanged in P10 rats, but was decreased in P21 rats when compared to age-matched saline groups and increased in adults when compared to both predrug responses and age-matched saline groups. CTOP significantly decreased mechanical threshold in adult rats when compared to predrug and age-matched saline responses. ^+^, ^++^*P* < 0.05, 0.01 compared to predrug responses. ^∗^, ^∗∗∗^, ^∗∗∗∗^*P* < 0.05, 0.001, 0.0001 compared to age-matched drug groups. ^##^, ^###^, ^####^*P* < 0.01, 0.001, 0.0001 compared to drug-matched age groups.

Baseline EMG activity in P10, P21, and adult rats were not significantly different (see Figs. 2A, B and 3A, B).

### 3.2 Spinal μ-opioid receptor activation causes a decrease in nociceptive responses in all ages

As neonatal animals have significantly lower mechanical thresholds than older animals, different ranges of stimuli were used in the different age groups. Fig. 2A and B shows typical raw EMG traces evoked by mechanical stimulation of the plantar hind paw using vFh in each age group before and after spinal administration of DAMGO (Fig. 2A) and CTOP (Fig. 2B). Direct application of DAMGO (30 ng) onto the L4-5 spinal cord of lightly anaesthetised rats (n for P10 = 4, P21 = 8, adult = 5) produced a reduction in spinal reflex excitability [2-way ANOVA, drug × age interaction: F(4, 135) = 4.43, *P* < 0.01; Fig. 2C]. Significant reductions in spinal excitability were observed at all age groups between saline- and DAMGO-treated animals (*P* < 0.0001 for P10 and adults and *P* < 0.001 for P21 rats). The degree of inhibition observed was significantly greater in the P10 rats compared to adults (P10 = 3.97 ± 0.97% vs adult = 50.76 ± 8.79%, *P* < 0.01), illustrating the greater potency of opioidergic ligands in younger animals. The MOR antagonist CTOP (120 ng) applied spinally had no effect on reflex excitability in all ages (n for P10 = 4, P21 = 7, adult = 7) when compared to saline or to predrug responses [P10: F(3, 9) = 3.82, *P* = 0.51; P21: F(3, 18) = 0.76, *P* = 0.53; adult: F(3, 18) = 1.39, *P* = 0.28; Fig. 2C].

Changes in reflex excitability were also accompanied by parallel changes in mechanical threshold (Fig. 2D). Mechanical thresholds significantly increased after DAMGO was applied spinally [2-way ANOVA, drug × age interaction: F(4, 135) = 5.70, *P* < 0.01; Fig. 2D]. Comparisons between the different drug treatment groups showed that DAMGO significantly increased thresholds compared to saline in P10 and P21 rats (*P* < 0.0001), but not in adults. As with changes in EMG excitability, comparisons between the age groups showed that DAMGO-mediated increases in threshold were larger in P10 and P21 rats when compared to DAMGO-treated adults (both *P* < 0.01; Fig. 2D). Spinal application of CTOP had no effect on mechanical threshold at any age when compared to saline-treated groups or predrug responses, but in P10 and P21 animals, post-CTOP responses were significantly different from post-DAMGO responses (*P* < 0.0001; Fig. 2D).

### 3.3 Intra-PAG DAMGO facilitates nociceptive responses in immature rats but inhibits them in adults

Previously, we have shown that DAMGO applied to the RVM is pronociceptive in P21 rats whilst being analgesic in adults [29]. The PAG is the major source of afferent input to the RVM and we were therefore interested in investigating whether this excitatory effect of DAMGO was also present in the immature PAG. Fig. 3A and B shows typical raw EMG traces evoked by mechanical stimulation of the plantar hind paw using vFh in each age group before and after intra-PAG microinjection of DAMGO and CTOP, respectively. Administration of DAMGO (30 ng; n for P10 = 4, P21 = 6, adult = 7) and CTOP (120 ng; n for P10 = 4, P21 = 7, adult = 5) produced differential responses in the different age groups with respect to reflex excitability [2-way ANOVA, drug × age interaction: F(4, 126) = 17.14, *P* < 0.0001; Fig. 3C] and mechanical thresholds [2-way ANOVA, drug × age interaction: F(4, 126) = 84.89, *P* < 0.0001; Fig. 3D]. In P10 rats, neither DAMGO nor CTOP had any effect on spinal excitability when compared to saline-treated animals or predrug responses [DAMGO: F(3, 9) = 3.32, *P* = 0.07; CTOP: F(3, 9) = 1.52, *P* = 0.28]. DAMGO, however, significantly facilitated spinal excitability in P21 rats (*P* < 0.001 vs saline; *P* < 0.05 vs predrug responses; Fig. 3C) and this was accompanied by a significant reduction in mechanical thresholds when compared to saline-treated rats (*P* < 0.0001 vs saline; Fig. 3D). The comparisons of the effects of intra-PAG DAMGO between P21 and both P10 and adult rats showed that DAMGO is pronociceptive in P21 rats only. Spinal reflex excitability after DAMGO injection into the PAG in P21 was significantly higher when compared to P10 and adult rats (*P* < 0.001 vs P10; *P* < 0.0001 vs adults; Fig. 3C). Mechanical threshold was also significantly lower in P21 when compared to adults (*P* < 0.0001; Fig. 3D).

### 3.4 Tonic opioidergic tone in the PAG of adults is absent in younger rats

CTOP (120 ng) significantly increased spinal excitability in adult rats (*P* < 0.0001 vs saline and DAMGO; Fig. 3C). In P21 rats, reflex excitability was increased compared to predrug responses only (*P* < 0.05; Fig. 3C), and this effect was small (100% vs 113.2 ± 6.85%). No effect was observed in P10 when compared to saline-treated age-matched controls or predrug responses. The increase in adult spinal excitability was accompanied by a significant reduction in mechanical threshold when compared to saline (*P* < 0.05) or changes in threshold in P10 or P21 rats (both *P* < 0.01; Fig. 3D). These data show that MOR-mediated signalling in the PAG produces differential responses over postnatal development. At P21, but not P10, DAMGO in the PAG is pronociceptive, whereas it is inhibitory in adult rats. Furthermore, antagonising MOR has significant effects in adult rats, indicating the presence of a tonic opioidergic tone within the mature PAG.

### 3.5 Transcription of genes encoding endogenous opioid peptides and receptors undergo significant postnatal alterations

The switch from net facilitatory to tonic inhibitory output in opioidergic signalling within the PAG over postnatal development may reflect underlying changes in the expression of MOR and opioidergic peptides. To test this hypothesis, TaqMan RT-PCR was employed to assess whether expression of endogenous opioidergic targets are changed during the postnatal period. TaqMan RT-PCR for POMC (pro-opiomelanocortin, precursor peptide of β-endorphin), ENK, and MOR (MOR-1A) was performed in the PAG and spinal cord of P10, P21, and adult rats.

**Fig. 4:**
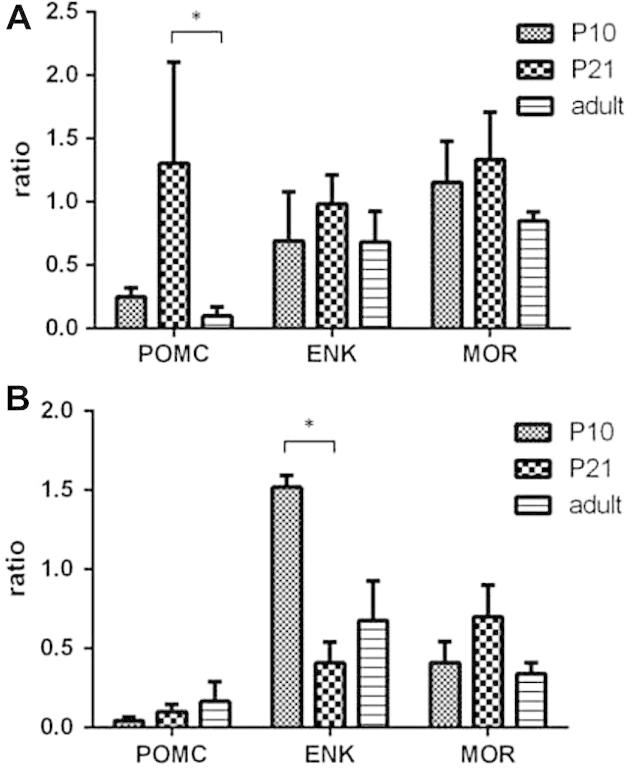
Transcription of genes encoding endogenous opioid peptides and receptors during postnatal development. (A) mRNA transcript numbers of POMC (pro-opiomelanocortin), ENK (enkephalin), and MOR (μ-opioid receptors) in the vPAG (ventral periaqueductal grey). There were no changes in ENK and MOR transcription levels between the ages. There was a significant increase in POMC mRNA transcript numbers at postnatal day (P)21; in particular, there were more POMC in P21 when compared to adult (^∗^*P* < 0.05). (B) mRNA transcript numbers of POMC, ENK, and MOR in the DH (spinal cord dorsal horn). There were no changes detected with either POMC or MOR. There was a significant increase in ENK mRNA transcript numbers as the animals aged, and it was highest at P10 (^∗^vs P21). Data presented in both A and B were expressed as mean ± SEM of the ratio of transcript number of target gene divided by transcript number of glyceraldehyde 3-phosphate dehydrogenase (GAPDH; reference gene).

Here we report significant changes in POMC mRNA copies in the vPAG between the 3 ages [one-way ANOVA, F(2, 16) = 3.65, *P* ⩽ 0.05; Fig. 4A]. In particular, posttests revealed there were significantly more POMC mRNA copies in P21 vPAG when compared to adult rats (*P* < 0.05). There were no changes in POMC mRNA copy numbers in the dorsal PAG (dPAG) throughout postnatal development (data not shown). Copy numbers of mRNA for ENK or MOR1A within the vPAG or the dPAG (data not shown) were not significantly different between the ages.

Transcription of ENK mRNA in the DH undergoes significant postnatal refinement [one-way ANOVA, F(2, 7) = 8.04, *P* < 0.05; Fig. 4B]. We found highest ENK mRNA copies in P10, and lowest at P21 (*P* < 0.05 vs P10). There were no significant age-related changes in ENK mRNA copy number in the ventral horn (data not shown), there were also no changes in MOR mRNA copy numbers in either the DH or the ventral horn (data not shown).

### 3.6 Age-dependent changes in the expression of POMC, ENK, and μ-opioid receptors within the PAG

**Fig. 5:**
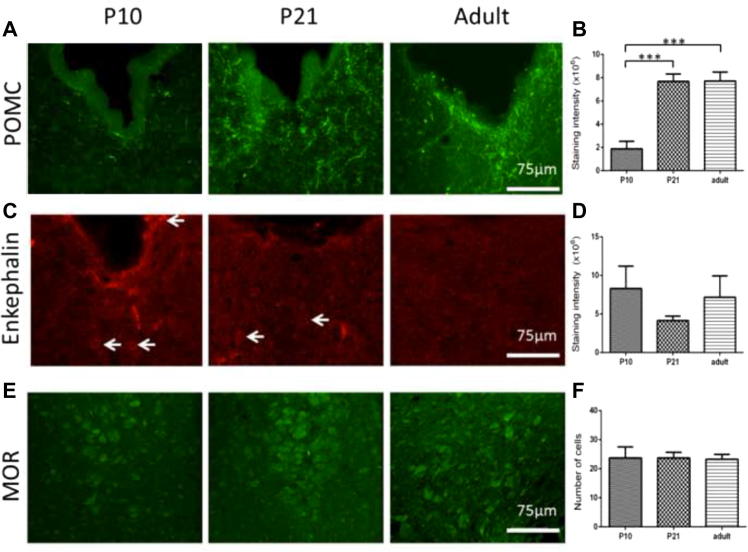
Immunohistochemical expression of opioid peptides and receptors in the vPAG (ventral periaqueductal grey) during postnatal development (A) Epifluorescent images showing POMC (pro-opiomelanocortin) immunoreactive fibre staining of the ventral PAG (vPAG) in postnatal day (P)10, P21, and adult animals. (B) A histogram showing quantified staining intensity of POMC in the vPAG of the 3 ages (measured by intensity function ImageJ), which increased dramatically as the animals aged (^∗∗∗^*P* < 0.001 vs P21 and adult). (C) Enkephalin immunoreactivity in the vPAG of P10, P21, and adult animals. (D) Staining intensity of enkephalin-positive fibres did not vary between the ages. (E) MOR (μ-opioid receptor) staining in the vPAG, the location was more ventral when compared to POMC and enkephalin images because that was where we found most MOR immunoreactive cells. (F) Cell counts for MOR positive cells in the vPAG, no changes were detected between the ages. All images shown in A, C, and E were imaged with a 40× objective with a confocal head.

Immunoreactivity for POMC was found in both the dPAG and the vPAG in all ages tested. Since there were no significant differences in POMC, ENK, and MOR immunoreactivity in the dPAG between the ages, these data are not shown. At P10, fibre staining of POMC can be observed in the vPAG, which became more restricted to the ventral side of the aqueduct at later ages. Staining intensity within the vPAG was statistically higher the older animals became [one-way ANOVA, F(2, 9) = 23.91, *P* < 0.001; Fig. 5B]. Posttests revealed that POMC immunoreactivity was the lowest in P10 (P10 vs adult and P21 both *P* < 0.001; Fig. 5B). Both ENK immunoreactive fibres and cells (indicated by white arrows; Fig. 5C) were found. Since only fibre staining could be found in the majority of sections investigated, staining intensity was measured, but no statistically significant changes in staining intensity were detected (Fig. 5D). MOR staining was observed in all the regions tested at all ages, but particularly in the more ventral parts of the vPAG (Fig. 5E). Since MOR immunoreactivity was found in cells, we quantified our data here by cell counting. There are no significant changes in the number of MOR-positive staining between the ages in the vPAG (Fig. 5F).

### 3.7 Age-dependent changes in the expression of POMC, ENK, and μ-opioid receptors within the DH

**Fig. 6:**
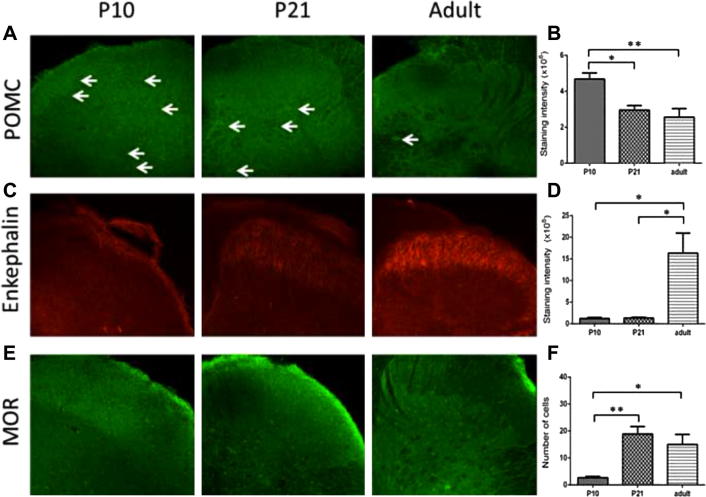
Immunohistochemical expression of opioid peptides and receptors in the DH (spinal cord dorsal horn) during postnatal development. (A) POMC (pro-opiomelanocortin) immunoreactivity in the dorsal horn in postnatal day (P)10, P21, and adult rats. White arrows depict where cell staining was found. Interestingly, fibre staining was also observed in the superficial dorsal horn (lamina I) of adult rats, but not in the younger ages. (B) Since both cell and fibre staining were observed, staining intensity was used to quantify the immunoreactivity of POMC in the DH. Quantified staining intensity for POMC in the DH significantly decreased as the animals aged, with highest immunoreactivity found at P10 (^∗^*P* < 0.05 vs P21; ^∗∗^*P* < 0.01 vs adult). (C) Enkephalin immunoreactivity in the DH was restricted to the superficial laminae and only fibre staining was observed. (D) Quantified staining intensity for enkephalin illustrate an age-dependent increase in immunoreactivity, which was highest at adult (^∗^*P* < 0.05 vs P21 and P10). (E) MOR (μ-opioid receptor) immunoreactivity in the DH, cell staining was found throughout the superficial and deeper laminae in all ages. (F) Cell count of MOR staining in the DH, which showed a significant increase as the animals aged (^∗∗^*P* < 0.01 vs P21; ^∗^*P* < 0.05 vs adult).

In the DH, POMC-positive cell staining was seen in both superficial and deeper laminae of the DH. As the animals aged, staining became more localised to the superficial lamina, and fibre staining could be seen in lamina I of adult rats (indicated by white arrows, Fig. 6A). Overall, the intensity of staining decreased as the animals aged [F(2, 9) = 9.16, *P* < 0.01]. Posttests revealed that POMC immunoreactivity was the highest in P10 (P10 vs P21, *P* < 0.05; P10 vs adult, *P* < 0.01; Fig. 6B). ENK immunoreactivity in the DH significantly increased as the animals aged [F(2, 13) = 8.61, *P* < 0.01]. Posttests revealed that ENK immunoreactivity was the highest in adult rats (P10 vs adult and P21 vs adult, both *P* < 0.05; Fig. 6D). Most ENK fibre staining in the DH was observed in the superficial laminae (lamina I and II) in all of the ages, but immunoreactivity intensified as the rats reached maturity. Significantly more MOR immunoreactive cells were found in the DH of older rats when compared to P10 animals [F(2, 15) = 9.64, *P* < 0.01]. Posttests revealed that there was more MOR immunoreactivity in the adult and P21 when compared to P10 in the DH (*P* < 0.05 and *P* < 0.01, respectively; Fig. 6E).

## 4 Discussion

Supraspinal nociceptive processing in neonatal animals and humans is significantly different from adults [21], [35], [40]. As the system matures, activation of supraspinal nociceptive pathways become increasingly inhibitory, thus, a fine-tuned balance of facilitatory and inhibitory control of spinal excitability is formed [21], [28], [29]. Little is known about the influences of immature supraspinal pathways on spinal nociceptive processing or the factors that drive the development of supraspinal nociceptive pathways throughout normal maturation [38]. One reason for this gap in our knowledge is that mechanisms underlying the switch from facilitation to inhibition during development are likely to be multifaceted [45].

Here we have focused on opioidergic pathways within the PAG and spinal cord. POMC is the precursor molecule for β-endorphin, which is a major bioactive opioid peptide. Both ENK and MOR are known to play a role in supraspinal pain processing and are expressed and functional within the descending pain modulation circuit [6], [14], [39], [41], [47]. Although not an exhaustive list of opioid receptors and related peptides, we have chosen MORs because they are known to be central to the function of the PAG, are abundantly expressed within the region [27], [44], and play a significant role in pain modulation [9], [25], [56].

### 4.1 MOR activation in the PAG differentially modulates spinal excitability in an age-dependent manner

The PAG and RVM represent the major supraspinal centres controlling spinal excitability [6], [19], [20]. Here we have demonstrated that the endogenous opioidergic system within the PAG significantly changes in the postnatal period of the rat. Pharmacological activation of the MOR via DAMGO (30 ng/rat) in the PAG is pronociceptive in P21 rats, antinociceptive in adults, and lacks an effect in P10s. We have previously observed DAMGO-mediated spinal facilitation in the RVM [29]. The PAG exerts descending control to the spinal cord DH indirectly via the RVM [20], and the concurrent reversal of the predicted effects of DAMGO within both the PAG and RVM indicates that these structures undergo comparable postnatal modifications. The cellular mechanisms responsible for the DAMGO-mediated facilitation of spinal excitability are currently unknown and are the focus of on-going experimental research. There are several studies that report facilitatory effects of opioid agonists [15], [29], with the MOR particularly being shown to be able to signal via Gs rather than the typical Gi-protein second messenger systems [51]. The underlying mechanisms by which this biased signalling occurs are currently unknown, but have significant implications for the study of opioids in neurodevelopment and for the utility of these drugs as analgesic agents.

There are numerous reports of opiate-mediated inhibition of cellular activity and integrated pain responses within the adult PAG [32], [49], [52], but few investigating the activity of opioid peptides in younger rats. Recently, a study has demonstrated potent opioid-mediated antinociception elicited from the PAG, RVM, or dorsolateral pons in P3, P10, and P14 rats, which contradicts data presented here [4]. However, the differences between our studies in terms of assay of pain behaviour, modality of stimulus, and dose of DAMGO administered may explain some of these contradictions. Our study is performed in lightly anaesthetised rats, which are routinely used in studies investigating pain control in neonates [8], [21], [35], [55]. This experimental preparation removes confounding factors such as handling stress, novel testing environment, and perhaps most importantly, the effects of a protracted period of maternal separation, which are known to significantly affect pain responding in neonates via an opioid-dependent pathway [33], [34].

Our electrophysiological studies (MOR-mediated antagonism by CTOP) showed that within the PAG, tonic opioidergic control of neurotransmission exists in adult rats. Although a small increase in spinal reflex excitability was seen in P21 rats when CTOP was injected into the PAG, there were no significant differences between post-CTOP and postsaline responses in both reflex excitability and mechanical threshold. On the other hand, in adult rats, CTOP increased spinal nociceptive reflex excitability, and mechanical thresholds (P40 on Fig. 3D) were also significantly decreased in adult rats after the drug was injected into the PAG. These results suggest that supraspinal MORs are tonically active in the healthy, mature pain-modulation circuit. It is also evident that, in our anatomical studies, the expression of POMC in the PAG increased as the animals aged (Fig. 5A, B). Together, these physiological and anatomical data suggest that opioid-related activity in the mature PAG is higher when compared to neonates. Neonatal injury is known to induce changes in the functioning of pain modulation in later life [30], [54], [55] and increase opioidergic tone from the PAG [36]. Neonatal pain experience therefore shapes pain responding in adulthood, and supraspinal opioidergic systems are central to this process.

### 4.2 Activating MOR in the spinal cord is more efficacious in young vs adult rats

Application of DAMGO to the spinal cord produced profound analgesia in all ages tested. This effect of DAMGO was significantly greater in P10 pups when compared to adults (Fig. 2). These findings suggest that opioid-mediated signalling in the spinal cord is stronger in the younger rats when compared to adults, which is in agreement with findings from previously published studies [10], [11], [12], [42], [43]. Opioid receptors and related peptides are present on both primary sensory afferents and intrinsic neurons within the adult DH [16]. These are often co-localised with calcitonin-gene related peptide [1] and substance P [7]. In neonatal animals, MOR binding sites in the spinal cord are equally concentrated in the superficial and deeper laminae, and as animals age, their expression becomes refined to the superficial DH [2]. Calcium imaging in cultured dorsal root ganglia has shown that neonatal sensory neurones of all fibre types (C, Aδ, and Aβ) are sensitive to morphine, whereas only small-calibre fibres are sensitive in adult dorsal root ganglia [42]. It is perhaps this functional reorganisation of MORs that leads to the differences in sensitivity and selectivity of opioidergic actions in the spinal cord between neonatal and adult rats.

Our data failed to show a significant increase in mechanical threshold following DAMGO administration to the adult spinal cord. Baseline thresholds in adult rats are significantly higher than in either P21 or P10 rats [21], and a ceiling effect was observed whereby thresholds increased beyond the range of mechanical stimuli available to us. It should be noted that spinal excitability in adults was significantly decreased.

### 4.3 Alterations in the transcription and expression pattern of opioid peptides and receptors

The switch in PAG MOR-mediated activity may also reflect the developmental regulation of the underlying cellular expression and distribution of opioid receptors within the circuit, and/or epigenetic control of genes that code for downstream signalling proteins [45]. It has been suggested that sufficient levels of endogenous opioids are needed for the maturation of pain pathways during a critical period between P21 and P28 [29]. The endogenous opioid system has also previously been implicated to play a role in the regulation of cell growth and differentiation in the developing brain [58]. One explanation for the increase in expression of opioidergic target may be that MOR-mediated activity is necessary for synaptogenesis within the descending pain modulatory circuit. The majority of previous studies investigating the ontogeny of the opioid peptides have relied upon SYBR Green [29] or radioimmunoassay [47] assays, but both have lower spatial resolution compared to immunohistochemistry, and lack the sensitivity of TaqMan RT-PCR used in this study [23].

Both immunofluorescence and TaqMan RT-PCR illustrated that expression level of POMC was highest at P21, which agrees with our previously described opioid-dependent critical period in the maturation of top-down pain control [29]. The expression of MOR shows a similar trend: increasing numbers of MOR immunopositive cells were observed in the DH as the animals aged, and highest level of expression was found at P21. The significant increase in POMC immunoreactivity around P21 coincides with the critical period of the development of supraspinal pain pathways [29], whereby the output of activating these pathways changes from being primarily facilitatory to increasingly inhibitory.

Immunofluorescence data show that ENK significantly increased in the DH as the animals aged, but the TaqMan RT-PCR data show that mRNA copy numbers were the highest in P10 DH. Although one may expect the increase in mRNA expression to precede protein translation, this is an unlikely explanation for these discrepancies. Instead it should be remembered that tissue taken for TaqMan will not include the cell bodies of the cells whose ENK-positive fibres are so prominent in the adult DH. The origin of these fibres is currently unknown, yet it is likely that these fibres are primary afferent nerve terminals rather than terminals of descending fibres.

These results altogether show that the endogenous opioid system undergoes crucial refinements in the descending pain modulatory pathway during postnatal development.

## Conflict of interest statement

The authors declare that they have no conflicts of interest with any of the work presented in this manuscript.
